# Prioritizing Gene Cascading Paths to Model Colorectal Cancer Through Engineered Organoids

**DOI:** 10.3389/fbioe.2020.00012

**Published:** 2020-02-04

**Authors:** Yanyan Ping, Chaohan Xu, Liwen Xu, Gaoming Liao, Yao Zhou, Chunyu Deng, Yujia Lan, Fulong Yu, Jian Shi, Li Wang, Yun Xiao, Xia Li

**Affiliations:** ^1^College of Bioinformatics Science and Technology, Harbin Medical University, Harbin, China; ^2^Key Laboratory of Cardiovascular Medicine Research, Harbin Medical University, Harbin, China

**Keywords:** gene cascading paths, prioritizing, colorectal cancer, engineered organoids, random walk with restart

## Abstract

Engineered organoids by sequential introduction of key mutations could help modeling the dynamic cancer progression. However, it remains difficult to determine gene paths which were sufficient to capture cancer behaviors and to broadly explain cancer mechanisms. Here, as a case study of colorectal cancer (CRC), functional and dynamic characterizations of five types of engineered organoids with different mutation combinations of five driver genes (*APC, SMAD4, KRAS, TP53*, and *PIK3CA*) showed that sequential introductions of all five driver mutations could induce enhanced activation of more hallmark signatures, tending to cancer. Comparative analysis of engineered organoids and corresponding CRC tissues revealed sequential introduction of key mutations could continually shorten the biological distance from engineered organoids to CRC tissues. Nevertheless, there still existed substantial biological gaps between the engineered organoid even with five key mutations and CRC samples. Thus, we proposed an integrative strategy to prioritize gene cascading paths for shrinking biological gaps between engineered organoids and CRC tissues. Our results not only recapitulated the well-known adenoma–carcinoma sequence model (e.g., AKST-organoid with driver mutations in *APC, KRAS, SMAD4*, and *TP53*), but also provided potential paths for delineating alternative pathogenesis underlying CRC populations (e.g., A-organoid with *APC* mutation). Our strategy also can be applied to both organoids with more mutations and other cancers, which can improve and innovate mechanism across cancer patients for drug design and cancer therapy.

## Introduction

The well-known adenoma–carcinoma sequence model described a basic carcinogenesis mechanism of colorectal cancer (CRC) (Vogelstein and Kinzler, 2004; Brenner et al., 2014). The sequential genetic alterations of *APC, KRAS, SMAD4*, and *TP53* could recapitulate the key features in transition from normal to adenoma and to initiation and progression of CRC, which promoted the understanding of pathogenesis in CRCs (Powell et al., 1992; Drost et al., 2015; Chen et al., 2016). Mutations on these genes could deregulate driver pathways to confer selective growth advantages and further to drive colorectal carcinogenesis. Tumor suppressor gene *APC* acted as an antagonist of the WNT signaling pathway. The inactivating mutations of *APC* could initiate a benign adenoma by activating the WNT pathway (Powell et al., 1992; Roper et al., 2017; Takeda et al., 2019), which was proved by the upregulation of β-catenin driven by APC mutations (Matano et al., 2015). The follow genetic alterations in *KRAS, SMAD4*, and *TP53* further promoted the transition of adenoma to CRC by activating EGFR, P53 and TGF-β pathways (Drost et al., 2015; Chen et al., 2016). *KRAS* was reported to play driver roles during the progression from early to intermediate adenoma stages (Takeda et al., 2019). The activating mutations in *KRAS* could activate EGF signaling. The *SMAD4* and *TP53* mutations promoted the transition from adenoma to adenocarcinoma stages (Fearon and Vogelstein, 1990). *SMAD* mutations reduced the *SMAD* protein and inhibited TGF-β signaling pathway. The mutation in *TP53* could overexpressed a truncated *TP53* protein which made *TP53* lose tumor suppressor roles (Tang et al., 2019). However, due to the high heterogeneity of genetic alterations across CRC population, it was inefficient for these driver mutations to characterize the molecular mechanism of broad CRC patients. Prioritizing different gene cascading paths for directing sequential introduction of key mutations were the pressing problem.

Organoids, as an *in vitro* 3D models, could closely recapitulate genetic spectra of original tissues (Morizane et al., 2015). For example, tumor organoids closely recapitulated the molecular spectra in CRC (van de Wetering et al., 2015). Introducing key mutations into organoids other than cells could provide better manners to examine the influence of driver genes during cancer carcinogenesis. Directly targeting modification of cancer genes could produce cancer cells from the mouse primary cells or *in vivo* tissue (Ran et al., 2013; Heckl et al., 2014; Platt et al., 2014; Sánchez-Rivera et al., 2014; Xue et al., 2014). Driver gene-targeted engineered organoids could grow in hostile medium while normal intestinal organoids ceased proliferation. We summarized the recent studies modeling CRC using intestinal organoids with introducing driver mutations in *APC, SMAD4, KRAS, TP53*, and *PIK3CA* (Table S1) (Cooks et al., 2013; Onuma et al., 2013; Drost et al., 2015; Matano et al., 2015; Chen et al., 2016; Nakayama et al., 2017; O'Rourke et al., 2017; Riemer et al., 2017; van Lidth de Jeude et al., 2017). *APC* mutations activated WNT signaling and promoted the growth of intestinal organoids in medium lacking WNT signaling (Matano et al., 2015). Intestinal organoids with *APC* mutations developed into benign tumors after transplantation (O'Rourke et al., 2017). *SMAD4* mutation-targeted organoids could grew in condition without inhibitor of TGF-β receptor signaling that was essential for sustaining the growth of normal intestinal cells (Matano et al., 2015). Engineered organoids expressing *KRAS* mutations could expand in the condition withdrawing EGFR signaling (Matano et al., 2015). *TP53* mutations induced prolongation of activation of NF-kappaB signaling, and promoted inflammation-associated colorectal cancer (Cooks et al., 2013). *TP53* mutation-targeted organoids could recover in the condition of activation of *TP53* signaling pathway which can induce cell cycle arrest and apoptosis (Matano et al., 2015). Oncogenic *PIK3CA* could regulate cell motility though *AKT*, and *PIK3CA* mutations played key roles in reprograming glutamine metabolism in colorectal cancers (Hao et al., 2016). *PIK3CA* mutations could induce cell attachment and motility under cooperation of *CTNNB1* (Riemer et al., 2017). Oncogenic *PIK3CA* could regulate cell motility though *AKT*, and *PIK3CA* mutations played key roles in reprograming glutamine metabolism in colorectal cancers (Hao et al., 2016). Sequential introducing different combinations of these driver mutations could delineate the progression from normal epithelium to adenoma and carcinoma. Engineered organoids with *APC* and *KRAS* mutations grew into lager dysplasia without invasive features (Takeda et al., 2019), and further formed invasive submucosal tumor under condition of inhibited TGF-β signaling pathway (Chen et al., 2016; Takeda et al., 2019). These studies implied that engineered organoids with sequential introducing driver mutations could provide new clues to exploring developmental mechanisms of cancers. However, whether these engineered organoids were sufficient to capture broad cancer behaviors were still a challenge.

The transformation of normal cells to tumor cells was the dynamic dysregulated procession of cellular homeostasis, which was the requirement for the organism function normally (Rosenfeldt et al., 2013). The activity of biological functions could reflect the extent of homeostasis. Many functional activity-based methods were proposed to reveal the disease mechanisms (Lee et al., 2008; Gatza et al., 2010; Drier et al., 2013). The patterns of functional activity made tumor disease classification more precise and built subtype characterizations (Lee et al., 2008; Gatza et al., 2010). The function dysregulated scores characterized the deregulated extent of functions in individual samples (Drier et al., 2013). Measuring the difference of function activity among different cancer stages could help characterizing the dynamic progression of CRC.

**Figure 1:**
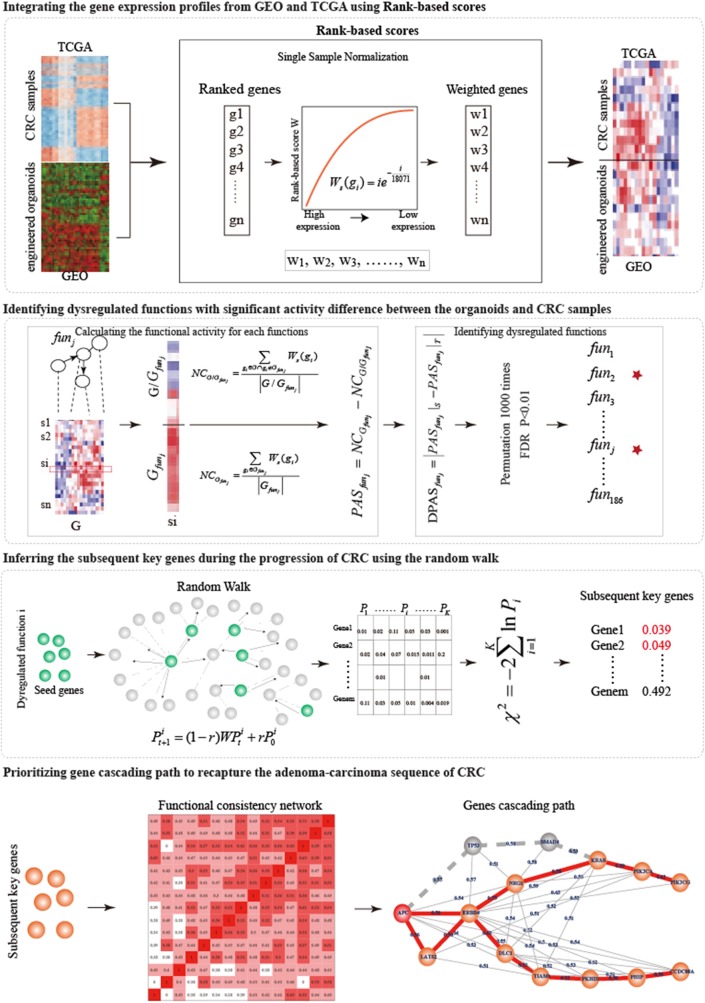
The overview of the integrative strategy for prioritizing gene cascading path.

In this work, from the single-mutant to quintuple-mutant engineered organoids, we dynamically characterized the function activities of hallmark signatures and measured the biological gaps between the engineered organoids and the CRC samples. An integrative strategy was designed to prioritize the gene cascading paths which could help us to understand the carcinogenesis mechanism of broad CRC patients with different profile of genetic alterations (Figure 1).

## Materials and Methods

### Data Collection and Processing

#### Gene Expression Profiles and Mutation Profiles of Colorectal Cancer

We downloaded the gene expression profiles (GSE57965) of adenoma and engineered organoids (Table S3), which contained five adenoma samples with *APC* mutation (A-organoid), 1 adenoma sample with genetic modification of *SMAD4* deletion (AS-organoid), 1 adenoma sample of genetic modification of knocking in *KRAS*^G12V^ (AK-organoid), 2 engineered human colon organoids carrying four gene mutations (*APC, KRAS*^*G*12*V*^, *SMAD4*, and *TP53*, AKST-organoids) and 1 engineered human colon organoids carrying five gene mutations (*APC, KRAS*^*G*12*V*^, *SMAD4, TP53*, and *PIK3CA*^*E*545*K*^, AKSTP-organoid) (Matano et al., 2015). The gene expression profile with 20,014 genes were obtained after removing probes corresponding to multiple genes and averaging the expression level of multiple probes of each gene.

We also downloaded the somatic mutation data (level 2) and gene expression profiles (RNA-seq) of colorectal cancer from the cancer genome atlas (TCGA). We extracted a mutation profiles which contained the samples with mutations in at least one of five genes (including *APC, SMAD4, TP53, KRAS*, and *PIK3CA*) and removed mutation types of silent, intron and 5'UTR. Finally, we obtained 103 samples with both gene expression profile and mutation profile (Table S3), in which 54 samples only with APC mutation, 40 samples only with mutations in both *APC* and *KRAS*, 3 samples with mutations only in both APC and *SMAD4*, 1 sample with mutations only in four genes (*APC, KRAS, SMAD4*, and *TP53*), and five samples with mutations of all of five genes.

#### KEGG Pathways and HPRD Protein Interaction Network

We downloaded the KGMLs of 222 human pathways from the Kyoto Encyclopedia of Genes and Genomes (KEGG) (Kanehisa and Goto, 2000). To get the topological information of these pathways, we got the corresponding undirected graphs of pathways and the degrees of genes in these pathways using the R package iSubpathwayMiner (Li et al., 2009). Only the pathways in which genes were connected with each other were kept. Finally, we obtained 186 pathways as the functions to characterize the biological gaps between organoids and cancer samples.

The protein interaction network was obtained from the Human Protein Reference Database (HPRD, version 9) (Keshava Prasad et al., 2009), which contained 9,617 genes and 39,240 interactions among these genes.

### Methods

We proposed an integrative strategy to prioritize the gene cascading path for directing CRISPR-Cas9 to construct colorectal cancer organoids (Figure 1).

#### Integrating the Gene Expression Profiles From GEO and TCGA Using Rank-Based Scores

To joint analysis of expression profiles from GEO and TCGA, we used the Rank-based scores (Amar et al., 2015) to normalize the expression profiles of engineered organoids and CRC samples. 18,071 common genes were detected by both GEO and TCGA. For each sample *s*, the expression values of 18,071 genes were sorted in the decreasing order. Rank of highest expressed gene was 1 and that of lowest one was 18,071. The rank *i* of gene *g* was transformed into rank-based score: $W_{s}\left(g_{i}\right)=ie^{-\frac{i}{18071}}$. The rank-based scores of genes in the samples were used to joint analysis.

### Identifying Dysregulated Functions in Biological Gaps Between Engineered Organoids and Corresponding CRC Samples

To investigate the potential driver capability of driver mutations, we characterized the biological distance from engineered organoids to CRC samples by identifying the dysregulated functions.

#### Functional Activity

Functional activity could measure the active status of biological functions in a specific sample (Bild et al., 2006). For each sample, we calculated functional activities of 186 functions using a Normalized Centroid shift method (Yang et al., 2012). For each function *j*, we classified the 18,071 genes (G) into two classes: genes within the function *j* (*G*_*fu*_*n*__*j*__) and the other genes (G/*G*_*fu*_*n*__*j*__). We calculated the average rank-based scores *NC*_*G*_*fu*_*n*__*j*___ and *NC*_*G*/_*G*__*fu*_*n*__*j*___, and then the activity score of function *j* (*FAS*_*fu*_*n*__*j*__) was calculated as the difference between *NC*_*G*_*fu*_*n*__*j*___and *NC*_*G*/_*G*__*fu*_*n*__*j*___.

$$\begin{array}{l} \;NC_{G_{fun_{j}}}=\frac{\underset{g_{i}\in G_{fun_{j}}}{\sum }W_{s}\left(g_{i}\right)}{\left|G_{fun_{j}}\right|}\\ NC_{G/G_{fun_{j}}}=\frac{\underset{g_{i}\in G\cap g_{i}\notin G_{fun_{j}}}{\sum }W_{s}\left(g_{i}\right)}{\left|G/G_{fun_{j}}\right|}\\ \;FAS_{fun_{j}}=NC_{G_{fun_{j}}}-NC_{G/G_{fun_{j}}} \end{array}$$

#### Identifying Dysregulated Functions With Significant Activity Difference Between the Engineered Organoids and CRC Samples

To measure the biological distance from engineered organoids (S) and corresponding CRC samples (T), we compared the activities of 186 functions between S and T. For each type of mutation combination, we calculated the average functional activities of each function, $FAS_{fun_{j}}^{s}$ and $FAS_{fun_{j}}^{T}$, for S and T. The $\text{DFA}{\text{S}}_{fun_{j}}=|FAS_{fun_{j}}^{s}-FAS_{fun_{j}}^{T}|$ measure the activity difference. To determine the significance of activity difference and identify dysregulated functions, the gene expression profiles of S and T were permuted 1,000 times, respectively. We re-calculated 1,000 random DFAS as described above. The significance *P* was calculated as the frequency in which random DFAS was larger than real DFAS. We identified the dysregulated functions as those at FDR = 0.01.

### Inferring Subsequent Key Genes During the Progression of CRC

The known driver mutations were inefficient to capture cancer behaviors and to broadly explain cancer mechanisms. Exploring the subsequent key genes of known driver mutations can improve the understanding of modeling CRC. We utilized Random walk with restart (RWR) (Köhler et al., 2008) to infer subsequent key genes during the progression of CRC for five types of organoids.

For each dysregulated function *k* obtained from a specific organoid, we reconstructed a biological network based on the pathway structure. We calculated the degrees of genes in the dysregulated function and selected the top 10% genes with the highest degrees as the seed genes which were the input of random walk. The seed genes were sowed into the protein interaction network. The information flow can restart from the seed genes with probability *r* in RWR (Köhler et al., 2008):

$$\begin{array}{l} P_{t+1}=\left(1-r\right)WP_{t}+rP_{0} \end{array}$$

where *r* was set to 0.7; $P_{0}$was the initial probabilities of genes, in which the probabilities of seed genes was 1n (n was the number of seed nodes) and others 0; $P_{t}$were the probabilities of genes at the *t*_*th*_ steps; *W* was the normalized transfer matrix of the protein interaction network; the random walk process reached the steady-state when the maximum difference between $P_{t+1}$and $P_{t}$was <10^−8^. The $P_{t+1}$ characterized the functional similarity of genes with seed genes. We randomly selected 1,000 sets of pseudo seed genes with the same size and re-performed random walk. For each gene *j* in the protein interaction network, the significance $P_{j}^{k}$ was calculated as the frequency in which random functional similarity was larger than real one. Finally, we combined the significance ($P_{j}^{k}$) of gene *j* calculated from all dysfunctional functions (*k* = 1……*K*) into a statistic *X* which follow the χ^2^ (2K) distribution:

$$\begin{array}{l} \chi^{2}=-2\overset{K}{\underset{k=1}{\sum }}\text{ln }P_{j}^{k} \end{array}$$

Where the K was the number of dysfunctional functions. The *P*(*X* ≥ χ^2^|*X* ~ χ^2^(2*K*)) represented the significance of genes. We considered genes with FDR ≤ 0.05 as subsequent key genes.

### Prioritizing Gene Cascading Paths to Recapture the Adenoma-Carcinoma Sequence of CRC

High tumor heterogeneity of genetic alterations in CRC made the well-known adenoma-carcinoma sequence explain a part of CRC patients, additional alternative gene paths were needed to interpret the development progression of more extensive CRC patients. Different patients with similar phenotype had different combinations of genetic alterations that tended to participate in same or similar functions. To prioritize gene cascading paths for each type of organoid, firstly, we calculated the functional coherence among the five known genes and the subsequent key genes (Wang et al., 2007), and constructed the functional consistency network at the threshold of 0.4. Then, a sparse functional consistency network was constructed by selecting two neighbors with highest functional consistency for each gene. Finally, using the well-known adenoma-carcinoma sequence model as the template, each gene cascading path was identified by starting from the mutant genes in the organoids and ending at the potential key gene showing the maximum shortest distance with mutant genes.

### Stepwise Comparison of Five Types of Organoids in the Activities of Hallmark Signatures

We compared the activities of 50 hallmark signatures among five types of organoids (including A-organoid, AS-organoid, AK-organoid, AKST-organoid, and AKSTP-organoid) in a stepwise way. For a pair of organoids, we identified the significant activation/inactivation of hallmark signatures in the organoid with more mutations by comparing with the other. The activities of 50 hallmark signatures were estimated using gene set enrichment analysis, and the activity differences between the pair of organoids were calculated. To measure the significance of activity differences, we permutated the transcriptomes of the pair of organoids 1,000 times, and recalculated 1,000 random activity differences of hallmark signatures. The significance of activation was calculated as the frequency in which random activity differences was larger than real one. And the significance of inactivation was calculated as the frequency in which random activity differences was smaller than real one. We identified the significant activation/inactivation of hallmark signatures at FDR ≤ 0.05.

## Results

### The Combination Mutation Patterns in Five Driver Genes Across CRC Populations

**Figure 2:**
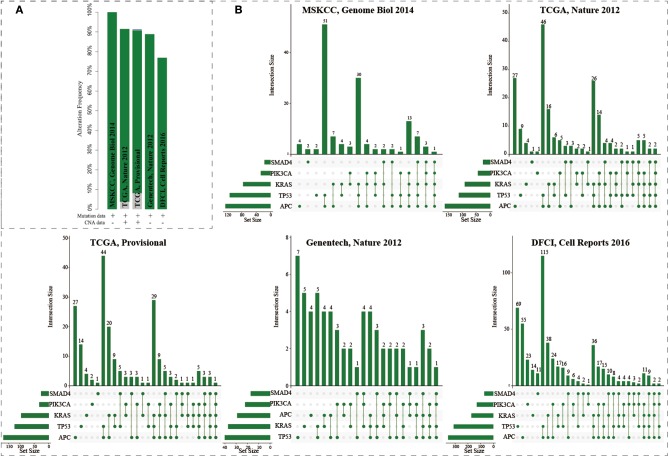
The mutation distributions of five driver genes in the five CRC populations. **(A)** The mutation frequencies of the five genes across five CRC populations. **(B)** Combination patterns of mutations in the five genes across CRC populations.

The mutations of five genes (including *APC, KRAS, SMAD4, TP53*, and *PIK3CA*) were reported to play driver roles in CRC progression. Five CRC populations in the cbioPortal were collected to investigate the mutation distributions of the five driver genes (Cerami et al., 2012; Gao et al., 2013). We found that these five genes showed high mutation frequencies ranging from 77 to 100% (Figure 2A). As a “gatekeeper” gene, *APC* mutations were extremely pervasive across CRC populations. Especially, the mutation frequency of *APC* reached up to 91% in MSKCC study (Figure S1 and Table S2). The mutation frequencies of *TP53* were 82, 53, 55, 56, and 43% across five CRC populations; 55, 42, 44, 51, and 28% for *KRAS*; 20, 20, 15, 31, and 21% for *SMAD4*; and 12, 14, 15, 24, and 10% for *PIK3CA*. The high frequencies of these five driver genes confirmed their core roles in the progression of CRC. Interestingly, only 0.72, 0.94, 0.45, 0% (0/72), 0% samples harbored the mutations of all five genes across the five CRC populations (Figure 2B). CRC samples harboring mutations in four genes only occupied 16.7, 5.7, 5.5, 8.3, and 3.9%, respectively. Most CRC samples (74.6, 65.1, 63.6, 58.3, and 54.1%) carried mutations of two or three genes. And the most common combination of mutations was observed between *APC* and *TP53*. These results further showed CRC was a highly heterogeneous disease from genomic perspective. Different CRC patients harbored different combinations of genetic alterations. The mutation frequency of single driver gene was high while the co-occurrence frequency of the five driver genes was very low. These phenomenon implied that although the mutations of the five driver genes could explain the CRC pathogenesis well, which could only explain the progressive mechanism for a fraction of CRC patients, but the molecular pathogenesis of major patients remains unclear. There existed other gene paths or mutation combinations to drive CRC evolution.

### Functionally Characterizing Engineered Organoids Carrying Various Combinations of Driver Mutations

We collected the transcriptomes of five types of engineered organoids which expressed mutations of different combinations of the five genes from GSE57965. For each type of engineered organoid, we calculated the activities of 50 hallmark signatures from MSigDB and identified the hallmark signatures with significant activation or inactivation using gene set enrichment analysis (Subramanian et al., 2005; Liberzon et al., 2015). In A-organoid, epithelial mesenchymal transition was the most significantly activated development signature (Figure S2A, *P* < 0.001). The immune signatures [IL6- JAK-STAT3 signaling (*P* = 0.0012) and inflammatory response (*P* = 0.001)] also showed significant activation. Five of six proliferation signatures showed significant activation in AK-organoid, which contained G2M checkpoint (*P* < 0.001) and E2F targets (*P* < 0.001). In AKST- and AKSTP-organoids, the hypoxia and glycolysis signature showed significant activation. Notably, none of 50 hallmark signatures showed significant inactivation in AKSTP-organoid (Figure S2B), indicating AKSTP-organoid exhibited more cancer hallmarks. These results suggested that the introduction of the five driver genes in intestinal organoids could induce the activation of hallmark signatures.

### Dynamically Analyzing CRC Progression From A- to AKSTP-Organoids

**Figure 3:**
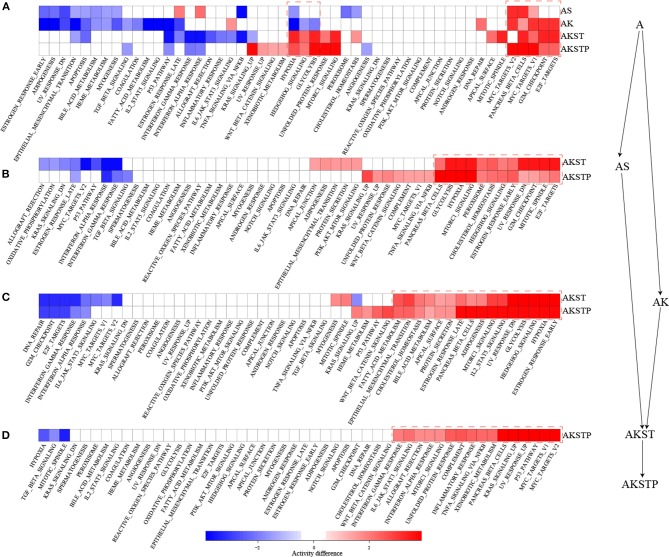
Dynamic activity analysis of 50 hallmark signatures from A-organoid (A) to AKSTP-organoid (AKSTP). **(A)** The significant activation (red) or inactivation (blue) of 50 hallmark signatures in AS-organoid (AS), AK-organoid (AK), AKST-organoid (AKST), and AKSTP-organoid (AKSTP) by comparing with A-organoid (A). **(B)** The significant activation (red) or inactivation (blue) of 50 hallmark signatures in AKST-organoid and AKSTP-organoid by comparing with AS-organoid. **(C)** The significant activation (red) or inactivation (blue) of 50 hallmark signatures in AKST-organoid and AKSTP-organoid by comparing with AK-organoid. **(D)** The significant activation (red) or inactivation (blue) of 50 hallmark signatures in AKSTP-organoid by comparing with AKST-organoid.

To further characterize the dynamic activities of hallmark signatures during sequential introduction of multiple driver mutations, we compared the activities of hallmark signatures between the five types of organoids. Compared with A-organoids, the other four types of organoids showed consistent activation of proliferation signatures containing G2M checkpoint and E2F targets (Figure 3A). Further, compared with AK- and AS-organoids, the AKST- and AKSTP-organoids consistently activated the hypoxia and glycolysis signature (Figures 3B,C). Compared with AKST-organoid, the AKSTP-organoid continued to enhance activation of proliferation signatures (MYC targets and P53 pathway) and immune signatures (Figure 3D). These dynamic analyses suggested that sequential introduction of these driver mutations gradually drove the activation of distinct hallmark signatures, and conferred the selective advantages to engineered organoids.

### Functionally Characterizing Combined Effects of the Five Driver Mutations Using TCGA CRC Patients

**Figure 4:**
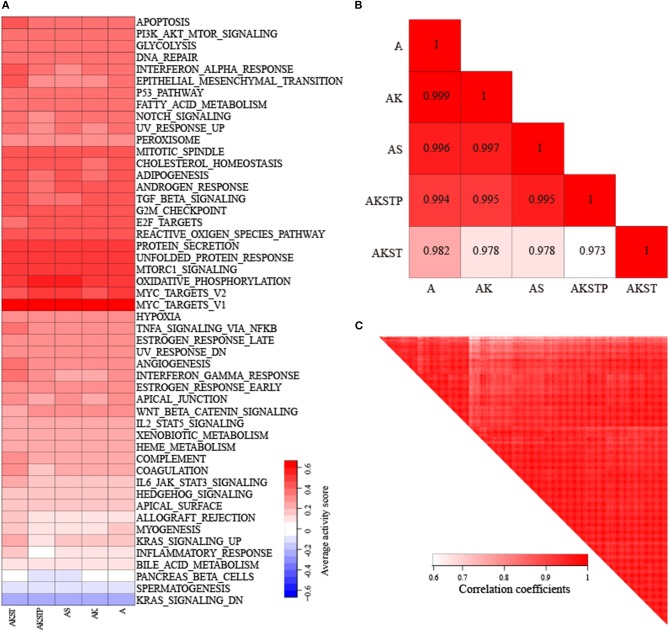
Highly consistent correlations of 50 hallmark signature activities across CRC samples with different combinations of mutations in five genes. **(A)** The average activity scores of hallmark signatures in five different groups of CRC samples. **(B)** The correlations of hallmark signature activities across five groups. **(C)** The correlations of hallmark signature activities across CRC samples.

We collected CRC samples with both expression and mutation profiles from TCGA. The mutations of the driver genes could influence gene expression levels of driver genes (*P* = 0.021 for *APC, P* = 0.0174 for *SMAD4, P* = 2.7e−5 for *TP53, P* = 0.0013 for *KRAS*, and *P* = 0.0183 for *PIK3CA*, Figure S3). According to the mutation status of the five driver genes, the 103 CRC samples were grouped into five groups (Table S3). To evaluate whether CRC samples with different combinations of driver mutations showed differential activities of hallmark signatures, we calculated the activities of hallmark signatures using single-sample GSEA for each CRC sample (Hänzelmann et al., 2013). For each group, average activities of hallmark signatures were calculated. We found that these five groups showed similar activated patterns (Figure 4A). The correlation coefficients of average activities ranged from 0.973 to 0.999 (Figure 4B). To further investigate whether the similar activated patterns also exited in all CRC samples, the correlation coefficients among all CRC samples were calculated. We found that all CRC samples still exhibited highly consistent correlation of hallmark signature activities in spite of different combinations of genetic alterations (Figure 4C). The results suggested that there existed additional driver genetic alterations contributing to development mechanism of broad CRC patients.

### Substantial Biological Gaps Between Engineered Organoids and Colorectal Cancer Tissues

We used the rank-based scores to integrate the expression profiles of engineered organoids and CRC samples. The result of principal components analysis showed that the expression pattern could distinguish the five types of organoids from TCGA CRC samples (Figure S4). To characterize the biological distance from the engineered organoids to CRC, we identified the dysregulated functions with significant activity difference between engineered organoids and their corresponding CRC samples at FDR = 0.01 against 1,000 permutations (Table S4).

**Figure 5:**
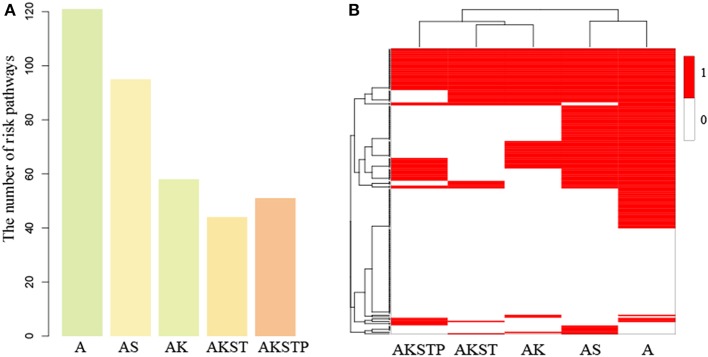
The dysregulated functions identified in the gaps between five types of organoids and CRC samples. **(A)** The number of dysregulated functions identified five types of organoids. **(B)** The binary heatmap of dysregulated functions across five types of organoids. (1 represents dysregulated functions, and 0 represents not).

For the A-organoids, we found that 65 of 186 functions showed no significant difference of functional activities by contrast to CRC samples, two of which *APC* participated in directly. For example, *APC* participated in the Wnt signaling pathway directly. In the A-organoids, the WNT pathway showed similar functional activity with the CRC samples with *APC* mutation (*P* = 0.015, Figure S5A). However, the Wnt signaling pathway showed significant activity difference (*P* = 0.008, Table S4) by comparing normal and CRC samples. These results suggested that *APC* mutation contributed the activation of Wnt signaling pathway, which was consistent with previous studies (Drost et al., 2015; Matano et al., 2015). Meanwhile, there were 121 dysregulated functions with significant activity difference. The MAPK signaling pathway showed significant activity difference between A-organoids and CRC samples (*P* < 0.001, Figure S5B). The number of functions showing similar activities between AK-organoids and corresponding CRC samples were up to 128, and the number of dysregulated functions decreased to 58. The RAS and MAPK signaling pathway showed similar activity between AK-organoids and CRC samples (*P* = 0.11 and *P* = 0.33, Figures S5C,D), suggesting the combination of *APC* and *KRAS* mutations enabled the activity of RAS and MAPK signaling pathway to reach the physiological state of CRCs. We also compared the function activity between AS-organoid, AKST-organoids, AKSTP-organoids and their corresponding CRC samples. We found that the number of functions with similar activity increased and the number of dysregulated functions decreased along with the number of genes mutations (Figure 5A and Table S4). These results gave a clue that combinations of multiple drive mutations approximated the organoids to CRC by activating or inactivating the activities of functions.

To characterize the step-by-step progression of CRCs from organoids engineered by introducing mutations, we compared the activity difference of 186 functions from five types of organoids. Firstly, we focused on the five functions including Wnt signaling pathway, RAS-MAPK signaling pathway, TGF-β signaling pathway, TP53 signaling pathway and PI3K signaling pathway, which were targeted by *APC, SMAD4, KRAS, TP53*, and *PIK3CA*, respectively. By comparing the normal and CRC samples, we found four functions including Wnt, RAS-MAPK, TP53 and PI3K signaling pathway showed significant differential activity(*P* = 0.008, *P* < 0.001, *P* < 0.001, and *P* = 0.003, Table S4). By introducing the mutations of corresponding genes, we found the significance of activity difference of four functions disappeared gradually (Table S5, FDR = 0.01). With the increasing number of mutated genes, the activity difference of these functions between organoids and CRCs tended to random state, suggesting the driver progression of key genes during carcinogenesis.

To further investigate the dynamic progression integrally, we clustered the organoids and the 186 functions based on the significance status of dysregulated functions. We found that A- and AS-organoids were a class, and AK-, AKST-, and AKSTP-organoids as a class (Figure 5B). *APC* mutation was a key gene for forming an adenoma. The adenoma still maintained the benign state after introducing *SMAD4* mutation. *KRAS* mutation made the adenoma canceration by dysregulating the activities of many functions, implying *KRAS* mutation played a key role during transformation from adenoma to CRC.

Among the 186 functions, 56 showed no significance of activity difference between any type organoid and CRCs. Twenty one functions also showed no significance between normal and CRC samples, indicating these functions may be essential functions for maintaining cell survival. However, the other 35 functions showed significant activity difference between normal and CRC samples, of which 16 functions were metabolism-related, implying the serious metabolic derangements have occurred from an adenoma. Meanwhile, we found that 27 functions showed significant activity difference between all of five types of organoids and CRCs, such as the PI3K signaling pathway, suggesting that additional key driver mutations were needed to transform the organoids to CRCs.

### Prioritizing Gene Cascading Paths Contributing to the Model of Colorectal Cancer Derived From Engineered Organoids

The five driver genes were not sufficient to make organoids approximate the physiological state of CRCs with features of metastasis and invasion (Matano et al., 2015). Meanwhile, due to tumor heterogeneity of CRCs, the mutations of five driver genes could explain development mechanisms of a part of CRC patients. Additional gene cascading paths were needed to explain the pathogenesis of broad CRC populations.

Using random walk to propagate information flow from dysregulated functions, we identified potential subsequent key genes for five types of organoids. At FDR = 0.05, we predicted 34, 89, 56, 4 potential key genes for A-, AS-, AK-, and AKST-organoids, respectively (Figures S6A,B and Table S6). For A- and AS-organoids, both *PIK3CA* and *KRAS* were identified, and *PIK3CA* was the top one gene identified from AK- and AKST-organoids, suggesting our method was able to identify key genes (Figure S6C). We also found that different organoids needed some common and specific potential genes to compete CRC progression (Figures S6B,C).

**Figure 6:**
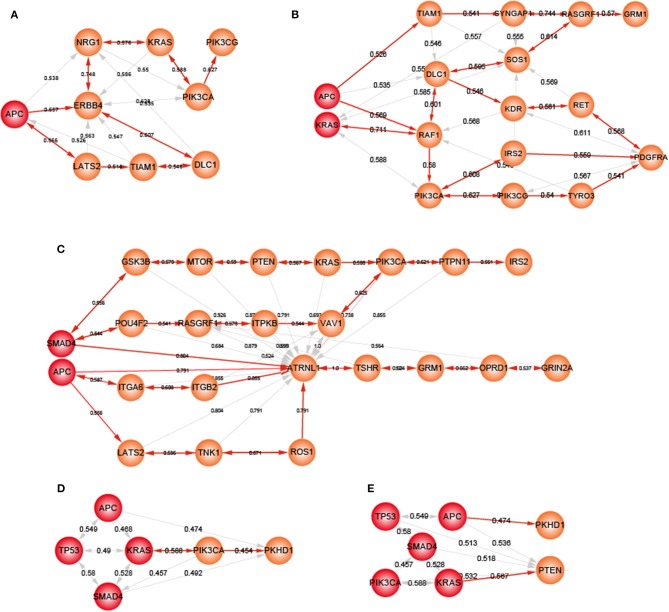
The potential gene cascading paths for five different organoids. Red node represents mutant genes in organoids, orange nodes for potential genes. **(A)** A-organoid. **(B)** AK-organoid. **(C)** AS-organoid. **(D)** AKST-organoid. **(E)** AKSTP-organoid.

Heterogeneity in genetic alterations across CRC populations indicated that different combinations of key genes contributed to the tumor progression through participating in similar functions. Prioritizing gene cascading paths for different organoids, which could perform analogical functions of five driver genes, could provide the interpretation of pathogenesis for broader CRC patients. Functional analysis showed the high functional coherence among the five driver genes. We calculated the function coherence among the potential genes and five known genes, and found that many potential key genes showed high functional coherence with the five known genes (Figures S7–S11). Thus, using the five driver genes as template, we prioritized cascading paths of key genes based on the function coherence to recapitulate the adenoma-carcinoma sequence model for different organoids (Figures 6A–E).

For A-organoids, two paths of potential key genes were predicted: one contained *APC, ERBB4, NRG1, KRAS, PIK3CA*, and *PIK3CG*, and the other contained *APC, ERBB4, LATS2, TIAM1*, and *DLC1* (Figure 6A). *ERBB4*, one of the ErbB receptor tyrosine kinases, showed the functional coherence of 0.56, 0.59, 0.63, and 0.57 with *APC, KRAS, PIK3CA*, and *TP53*, respectively, which also participated in cancer associated functions such as MAPK cascade, cell migration and cell proliferation. The colonic inflammation was limited by ErbB4 signaling through stimulating pro-inflammatory macrophage apoptosis (Schumacher et al., 2017). *ERBB4* itself could not induce tumor transformation of mouse colonocytes, while under the condition of colonocytes with mutant Apc and Ras, *ERBB4* enhanced the transformed phenotype both *in vitro* and *in vivo* (Williams et al., 2015). The increased co-expression of ErbB4-CYT-2 with KITENIN promoted the transition of colon adenoma to adenocarcinoma in tumor microenvironment of *APC* loss (Bae et al., 2016). *NRG1*, neuregulin 1, showed the functional coherence of 0.54, 0.58, 0.55, 0.58, and 0.51 with *APC, KRAS, PIK3CA, SMAD4*, and *TP53*, respectively. In the ERBB signaling pathway, *NRG1* could participate in cell migration and invasion by activating *ERBB4* and *KRAS*, and contribute to cell cycle and cell metabolism by activating *ERBB4* and *PIK3CA*. *NRG1* was methylated in tumors and the knockdown of *NRG1* could increase net cell proliferation (Chua et al., 2009). Paracrine NRG1/HER3 signals promoted CRC cell progression, and was associated with poor prognosis in CRC (De Boeck et al., 2013). *PI3KCG* was a critical switch between immune stimulation and suppression during inflammation and tumor growth (Kaneda et al., 2016). The silencing of *PIK3CG* contributed to inhibit the PI3K-Akt/PKB signaling system which was responsible for the tumorigenesis and progression of colorectal cancers (Semba et al., 2002). Thus, *ERBB4* and *NRG3* may replace *SMAD4* and *TP53* to form a new combination, together with *APC, KRAS* and *PIK3CA*, to form an alternative path underlying CRCs.

For ASKT-organoids, *PIK3CA* was ranked first, together with *APC, SMAD4, KRAS*, and *TP53*, which restored the known the adenoma-carcinoma sequence model of CRC (Figure 6D). ASKTP-organoids were capable to form the tumors while showed weak invasive behavior. Additional key genes were needed to complete the progression of CRC. *PKHD1* were the second potential key genes which showed function coherence of 0.47, 0.49, 0.48, 0.45, and 0.45 with *APC, SMAD4, KRAS, TP53*, and *PIC3CA*, respectively. The protein encoded by *PKHD1* harbored the structural features with hepatocyte growth-factor receptor and plexins which involved in regulation of cell proliferation and cellular adhesion and repulsion (Onuchic et al., 2002). Inhibition of *PKHD1* may control cell cycle via mTOR signaling pathway (Zheng et al., 2009), and induced cell apoptosis through PI3K and NF-κB pathways (Sun et al., 2011). We found that *PKHD1* showed high frequency of mutations in the CRC populations (from 8.9 to 11.8%, Figure S12). Previous studies showed that *PKHD1* was a candidate CRC gene by screening mutations in the consensus coding sequences profile, and was assigned to the function of cell adhesion with the first rank (Sjöblom et al., 2006). The germline mutations of *PKHD1* played a protective role in colorectal cancer (Ward et al., 2011). Thus, introduction of *PKHD1* mutations following the five driver genes may contribute to CRC invasion and metastasis.

## Discussion

The adenoma-carcinoma sequence was recognized as the mechanism model of CRC, in which mutations of *APC, KRAS, SMAD4, TP53*, and *PIK3CA* could sequentially drive CRC transformation. The sequential introduction of CRC genes was used to model colorectal cancer. These studies gave a clue that it is possible to investigate the CRC dynamic progression using engineered organoids. We proposed an integrative strategy to characterize the dynamic progression of CRC and prioritize gene cascading paths for directing subsequent introductions of key genes.

Dynamic analysis of activities of biological functions showed biological gaps between organoids and CRC tissues. The number of dysregulated functions dropped sharply with the number of mutations of key genes increasing. These results were consistent with previous studies (Drost et al., 2015; Matano et al., 2015), suggesting that our method could capture biological dynamics and characterize the CRC progression. The AKST- and AKSTP- organoids approximated the true CRC with corresponding mutations. However, there were still many dysregulated functions associated with tumor metastasis, such as cytokine-cytokine receptor interaction, ECM-receptor interaction, and adherent junction. Meanwhile, some tumor microenvironment associated functions including antigen processing and presentation, leukocyte transendothelial migration and chemokine signaling pathway were also in these biological gaps. The identified dysregulated functions may provide an explaining that AKST- and AKSTP-organoids without features of migration and invasion may be due to lacking of tumor microenvironment supporting invasion and metastasis. Additional driver mutations of key genes were needed to further identify to control these functions.

Through screening the genetic alteration profiles of CRC populations, the co-occurrence frequency of five CRC genes was low. Although the adenoma-carcinoma sequence of CRC was recognized, it only explained molecular mechanism in a fraction of CRC populations with mutations of all five genes. The genetic alterations of CRC populations showed high heterogeneity, implicating that other key genes were required for drawing the mechanism of colon carcinogenesis for most of CRC populations. Our method not only could characterize biological gaps between different types of organoids and their corresponding CRC samples, but also be able to predict key genes which followed the introduced key mutation to further shrink biological gaps. The potential sequential genes were identified for different types of organoids, which participated in important functions and pathways. For example, for the AK-organoids, 56 subsequent genes were predicted. Using functional enrichment, many cancer-associated functions, such as MAPK cascade, Ras signaling pathway, PI3K-Akt signaling pathway, positive regulation of cell migration and positive regulation of cell proliferation, were identified (Table S7). With the accumulation of published studies about CRC organoids and multidimensional omics data of organoids (Fumagalli et al., 2017; Newey et al., 2019; Ooft et al., 2019), our method could be used to identify more extensive gene paths and construct the landscape of molecular pathogenesis for CRC cancer. Sequential introduction of the mutations in gene paths may provide a new avenue for understanding the dynamic progression of CRC.

In summary, we developed an integrative strategy to capture the dynamic progression of CRC and prioritize gene cascading paths for understanding the mechanisms of wide CRC patients. Our approach also can reveal the dynamic transformation mechanism of other cancer types. This will provide a more detailed interpretation for molecular mechanisms of cancer which could help for drug design and cancer therapy.

## Data Availability Statement

Publicly available datasets were analyzed in this study. This data can be found here: GSE57965(https://www.ncbi.nlm.nih.gov/geo/query/acc.cgi?acc=GSE57965), TCGA(https://portal.gdc.cancer.gov/).

## Author Contributions

XL and YX designed and guided this work and LW supervised this work. YP, CX, LX, and GL participated in data processing, program implementation, and paper writing. YZ, CD, YL, FY, and JS contributed to data collecting and organized the figures and tables. All authors provided critical advice for the final manuscript.

### Conflict of Interest

The authors declare that the research was conducted in the absence of any commercial or financial relationships that could be construed as a potential conflict of interest.
