# Supplementary material for: Prioritizing Gene Cascading Paths to Model Colorectal Cancer Through Engineered Organoids
Source: Front Bioeng Biotechnol. 2020 Feb 4;8:12. doi: 10.3389/fbioe.2020.00012 (PMC7010597; doi:10.3389/fbioe.2020.00012)
Supplement: Supplementary file 4 [file Data_Sheet_1.pdf]

Supplementary Figures

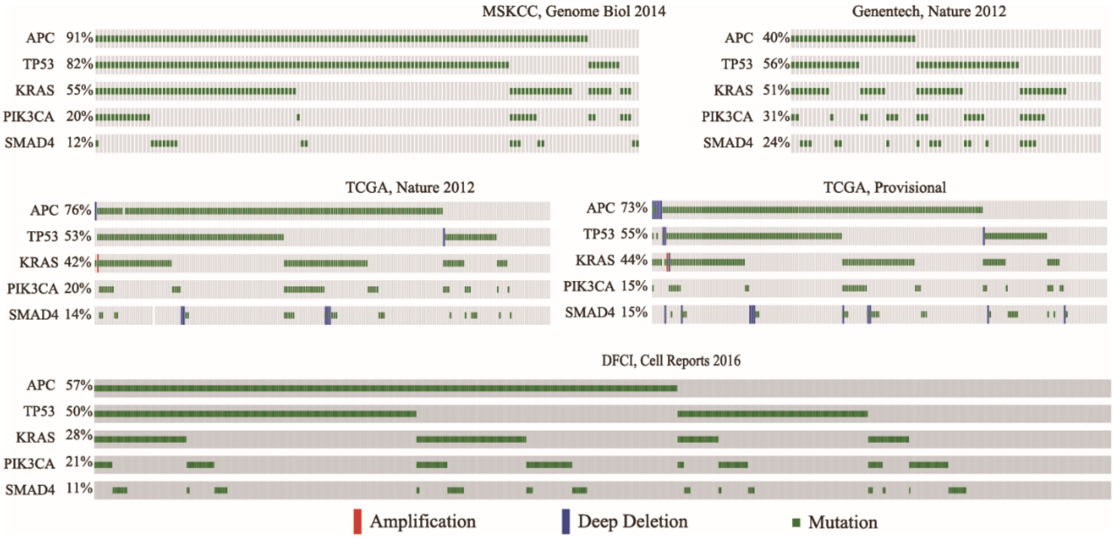

Supplementary Figure 1.The genetic alteration profiles of five genes in the CRC populations.

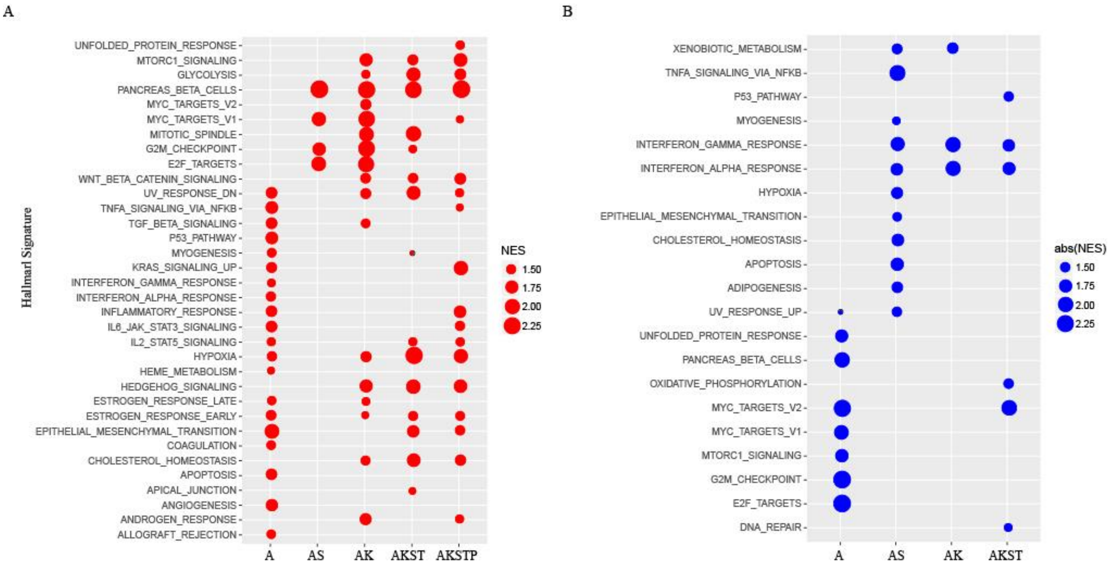

Supplementary Figure 2. The significant activated (A) or inactivated (B) hallmark signatures identified in five types of organoids.

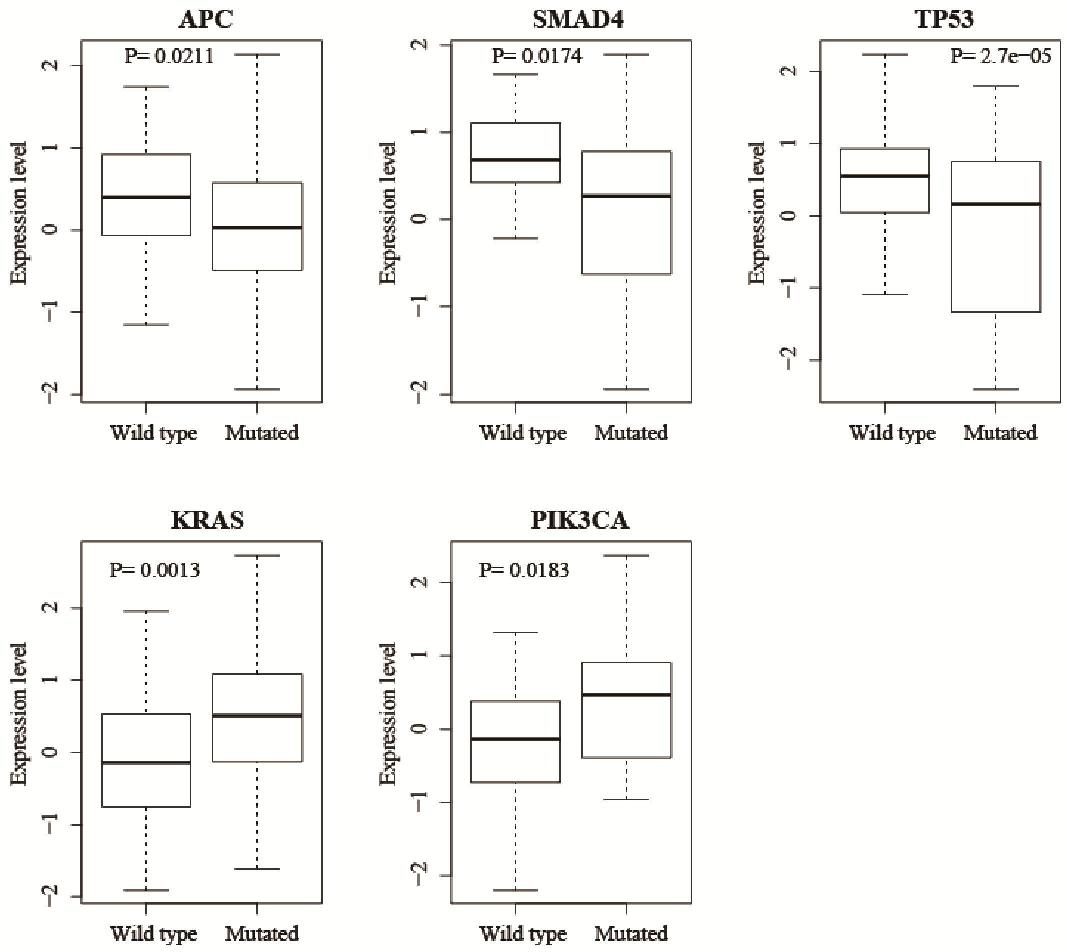

Supplementary Figure 3. The mutations influenced gene expression levels of driver genes.

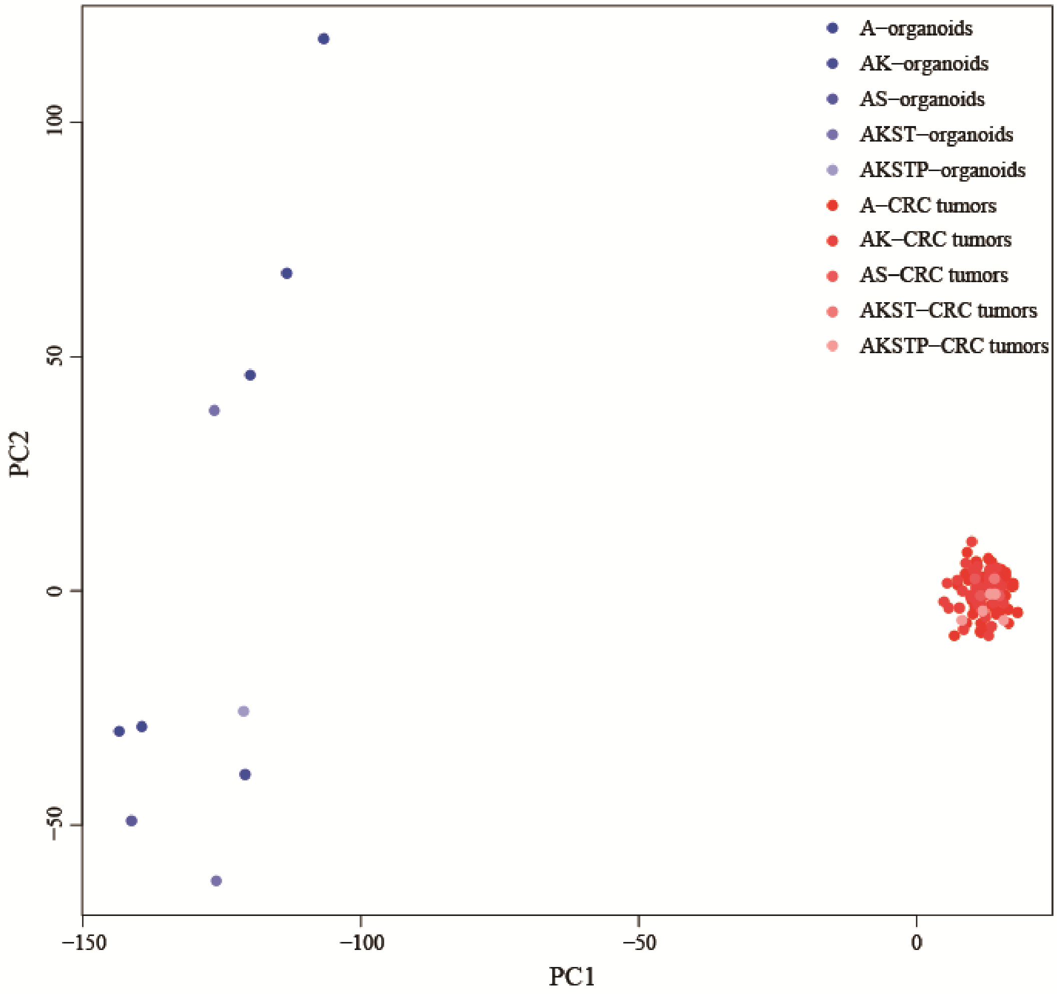

Supplementary Figure 4. Principal Components Analysis (PCA) on the normalized expression data from five types of organoids and TCGA CRC samples.

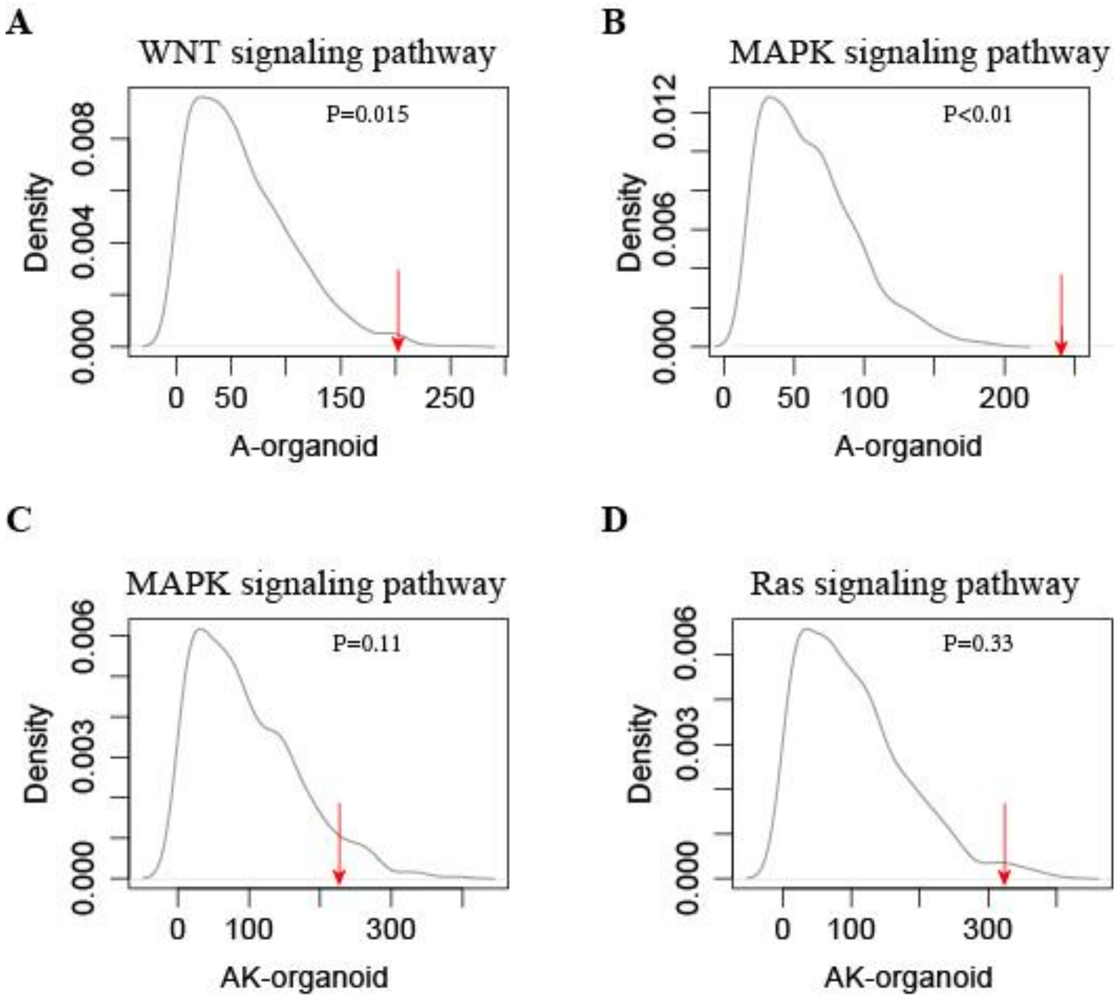

Supplementary Figure 5. The significance of activity difference of pathways between organoids and CRC samples. (A) and (B) for Wnt signalling pathway and MAPK signalling pathway between A-organoids and corresponding CRC samples. (C) and (D) for MAPK signalling pathway and RAS signalling pathway between AK-organoid and corresponding CRC samples.

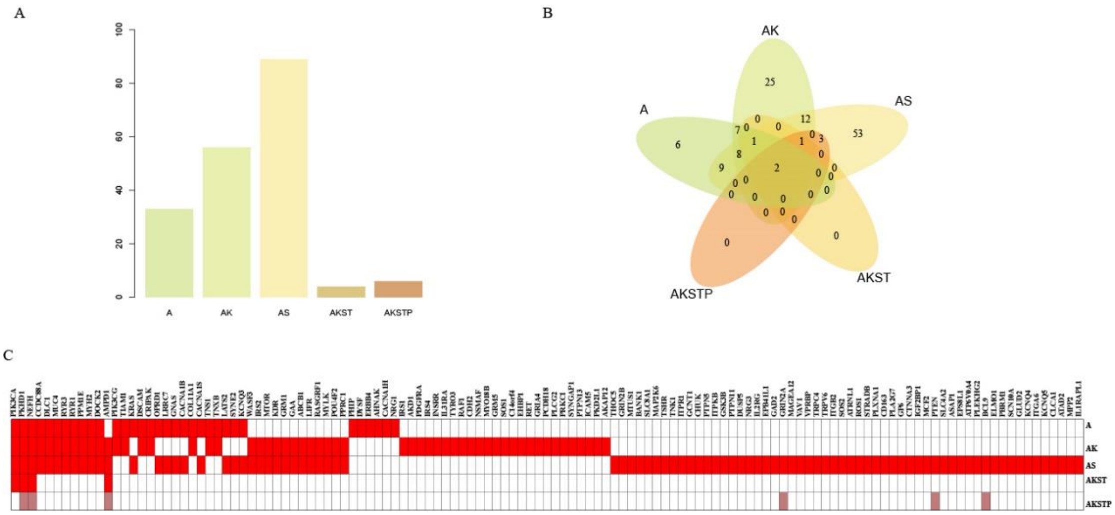

Supplementary Figure 6. The potential subsequent key genes identified for the five types of organoids. (A) The number of potential subsequent key genes. (B) The venn plot of potential subsequent key genes cross five types of organoids. (C) The heatmap matrix of subsequent key genes across five types of organoids, red at FDR=0.05, brown at P=0.05.

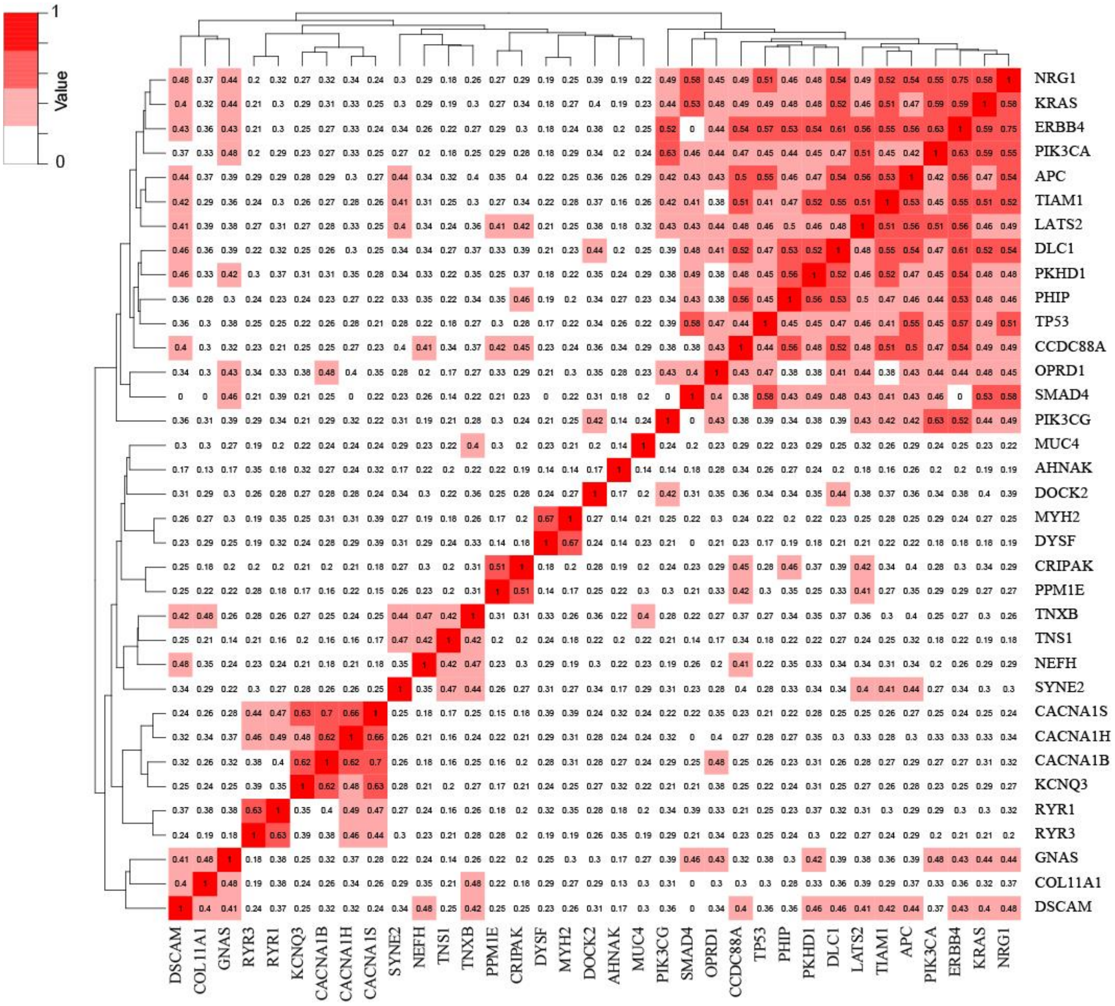

Supplementary Figure 7. The functional coherence among subsequent potential genes identified for A-organoid and five known driver genes.

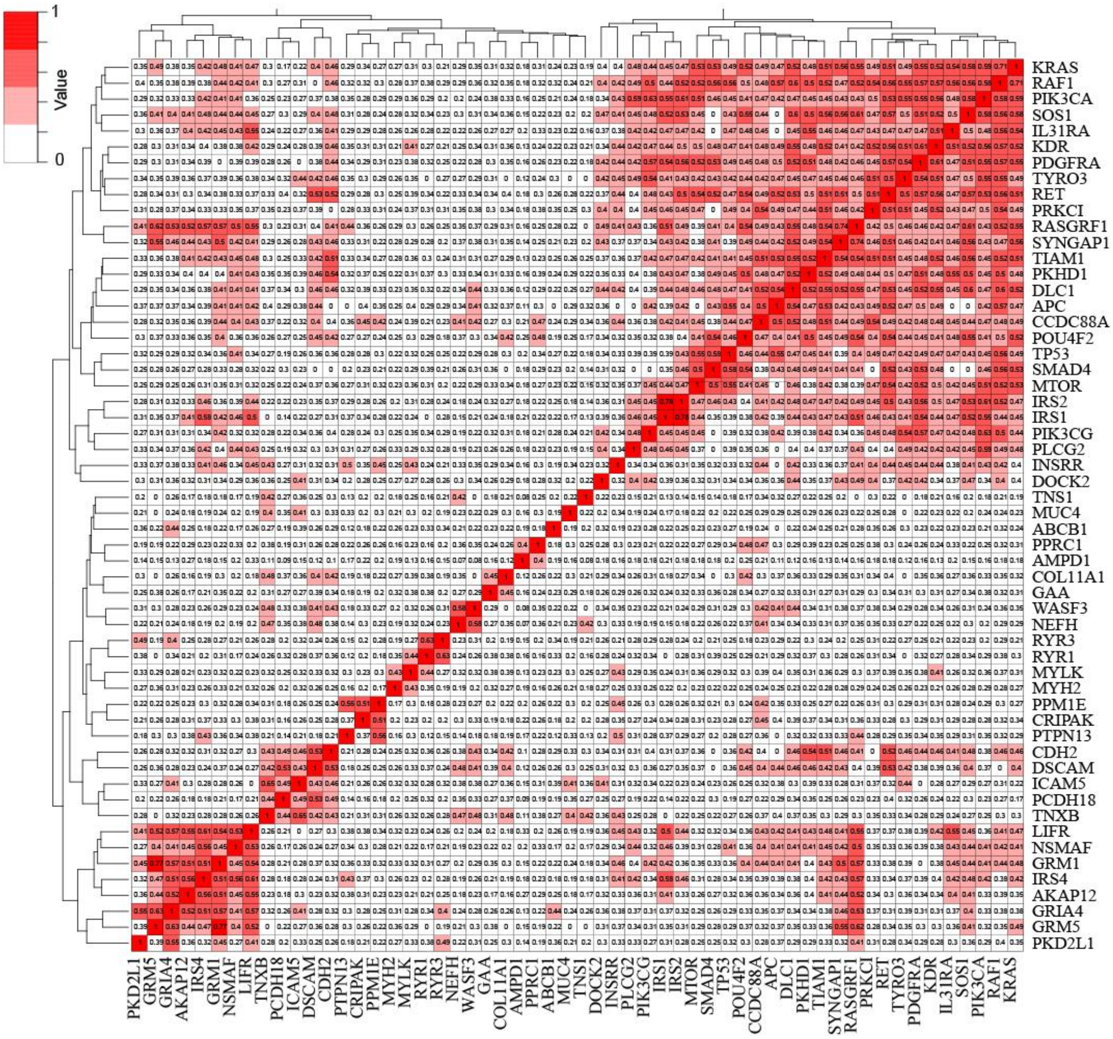

Supplementary Figure 8. The functional coherence among subsequent potential genes identified for AK-organoid and five known driver genes.

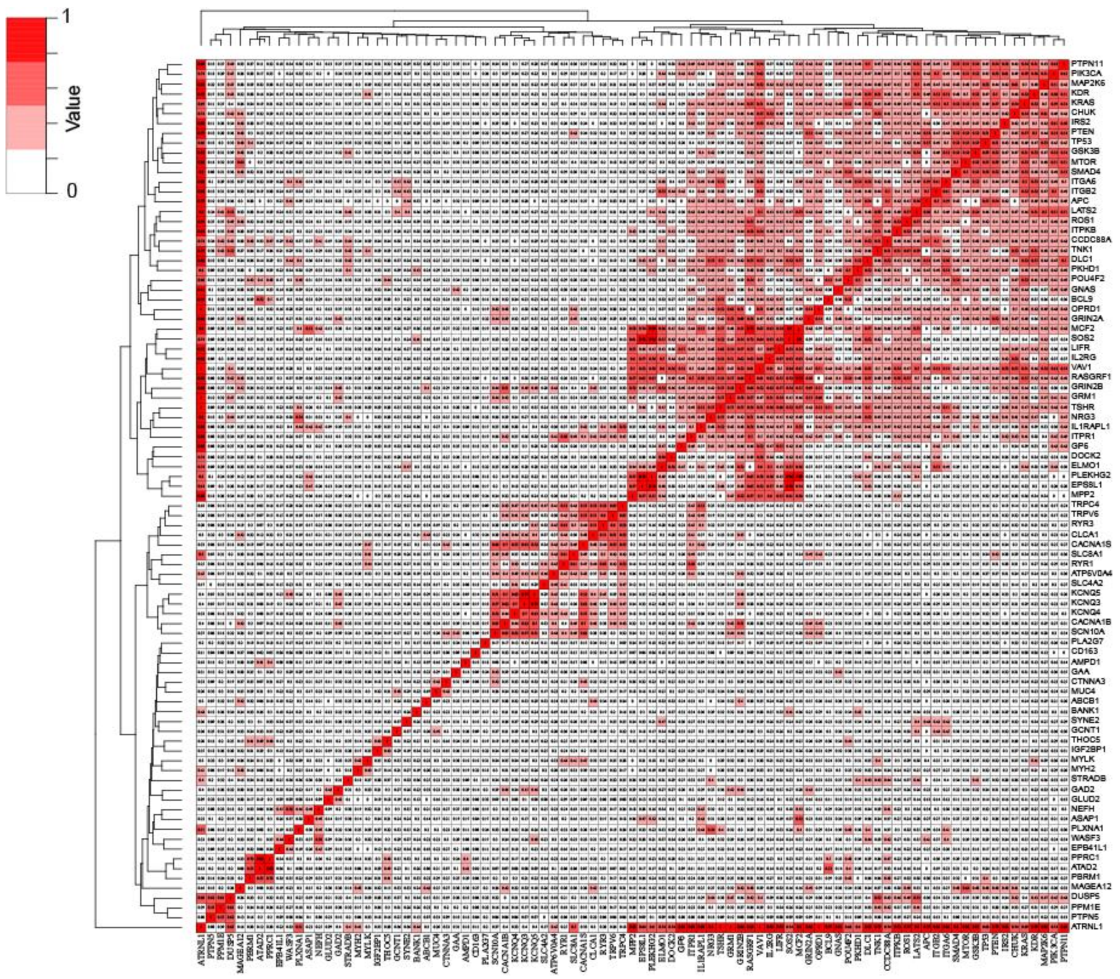

Supplementary Figure 9. The functional coherence among subsequent potential genes identified for AS-organoid and five known driver genes.

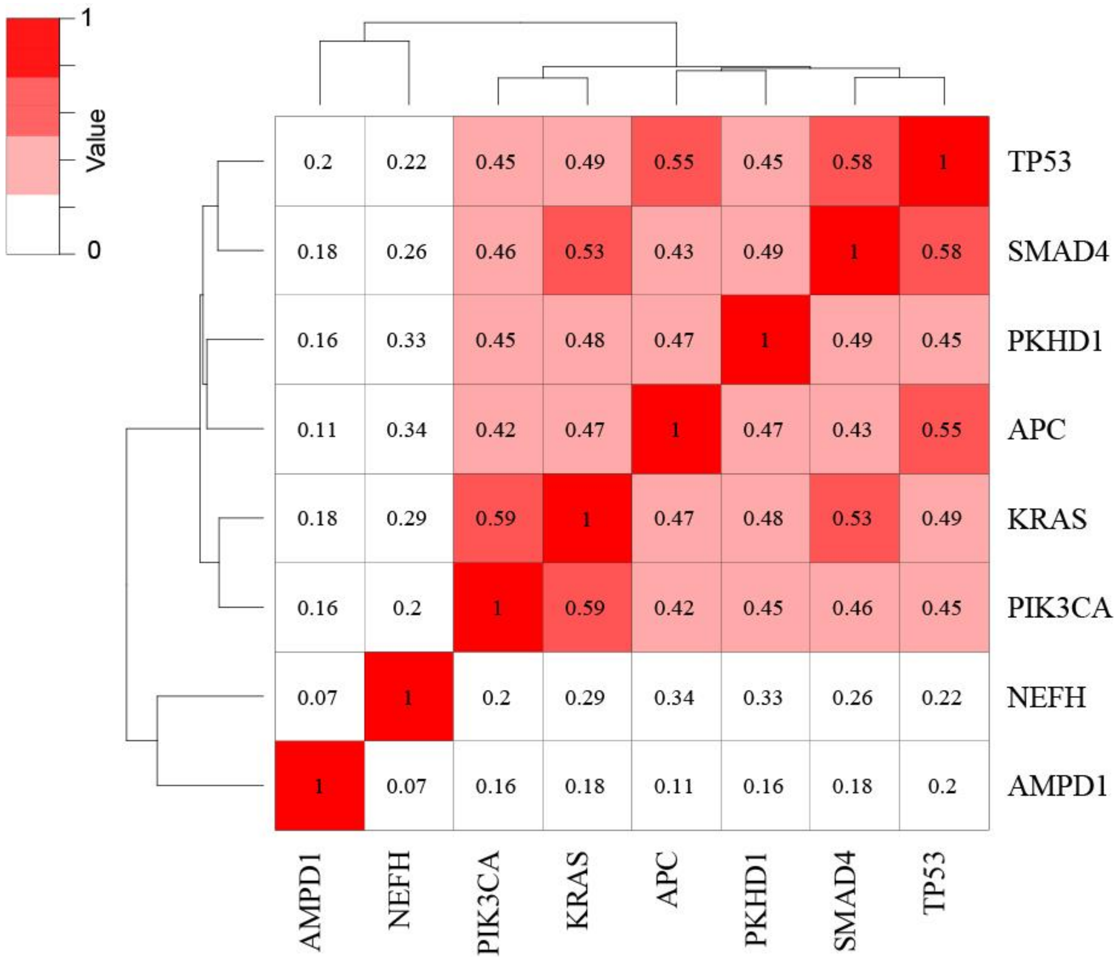

Supplementary Figure 10. The functional coherence among subsequent potential genes identified for AKST-organoid and five known driver genes.

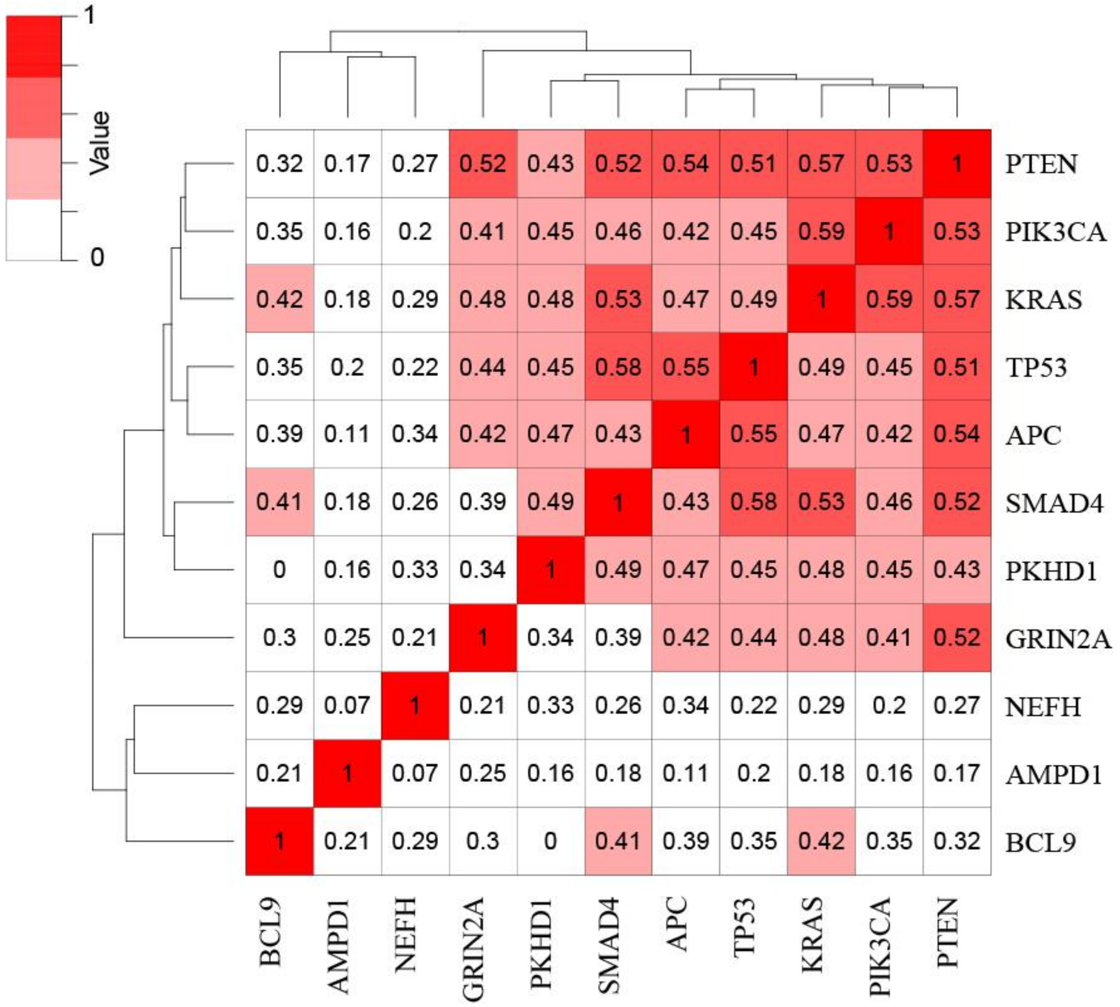

Supplementary Figure 11. The functional coherence among subsequent potential genes identified for AKSTP-organoid and five known driver genes.

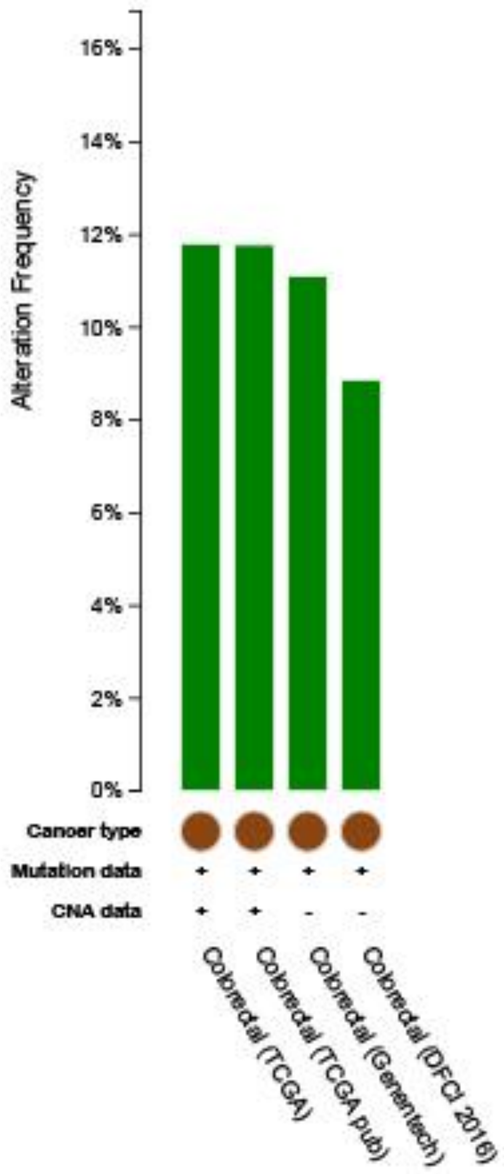

Supplementary Figure 12. The alteration frequency of *PKHD1* mutations in the CRC populations

Supplementary Tables

Supplementary Table 1. Summary about the different combinations of the five driver genes introduced in organoids

Supplementary Table 2. The mutation distribution of five genes across CRC populations.

Supplementary Table 3. The number of engineered organoids and CRC samples

Supplementary Table 4. The significance of activity difference for 186 functions between normal, organoids and CRC samples.

Supplementary Table 5. The significance of activity difference for functions targeted by five driver genes.

Supplementary Table 6. The potential subsequent key genes identified for the five types of organoids.

Supplementary Table 7. The significant functions and pathways enriched by the potential subsequent key genes identified for AK-organoids.

Supplementary Tables

Supplementary Table 1 Summary about the different combinations of the five driver genes introduced in organoids

Combinations	Organoids/cells	Pathways	phenotypes	Reference
APC	Human intestinal organoids[4, 13]<br>Mouse intestinal organoids[14]<br>hCECs[5]<br>genetically engineered mouse models (GEMMs) organoids [15]	WNT signalling pathway [4, 13, 14, 16]	Grew in medium lacking Wnt signaling [4, 13]<br>similar to *APC*-deficient adenoma[14]<br>Dysplasia[5]<br>benign tumors[15]<br>Hyperproliferation[20]	4, 5, 13-16, 20
SMAD4	Human intestinal organoids [13]	TGF-β signalling pathway [13]	Grew in medium lackingTGF-β receptor inhibitor [13]	13
KRAS	Human intestinal organoids [4]	RAS signaling[14]	Grew in medium without EGF and with EGFR inhibitor [4]	4, 14
TP53	Human intestinal organoids [13]<br>mouse intestinal organoids[17]	TP53 signalling pathway [4, 13]<br>NF-κB signalling [17]	Grew in medium with inhibitor of MDM2[13]<br>Chronic Inflammation and Inflammation Associated Colorectal Cancer[17]	4, 13, 17
APC, KRAS	Human intestinal organoids [4, 13]<br>Mouse intestinal organoids[14]<br>hCECs[5]	EGFR signaling[13]<br>angiogenesis[14]	Grew in medium without EGF[13] or lacking Wnt signaling [4]<br>larger dysplasia, large adenomas with invading the submucosa when TGF-β signaling pathway was inhibited[5]	4, 5, 13, 14
APC,TP53	Human intestinal organoids [4]<br>Mouse intestinal organoids[14]<br>Mouse *i*ntestinal polyps[18]		Extensive aneuploidy [4]<br>tumors on both sides of nude mice, Tumorigenesis from Organoids [14]<br>Submucosal invasion[18]	4, 14, 18
PIK3CA<br>CTNNB1	transgenic mouse[19]	PI3K signaling, glycolysis and oxidative phosphorylation [19]	induction of cell attachment and motility [19]	19
APC, KRAS, TP53	Human intestinal organoids [4]<br>genetically engineered mouse models (GEMMs) organoids [15]		Grew in medium lacking Wnt and R-spondin and with nutlin-3, visible nodules [4]<br>carcinomas with submucosal invasion [15]	4, 15

APC, KRAS PIK3CA	Human intestinal organoids [13]	EGFR signaling[13]	Grew independently of EGF signaling [13]	13
APC, KRAS, TP53, SMAD4	human intestinal organoids[4, 13]		visible tumor, adenoma histology [13] Grew without all niche factors, tumours with proliferative and invasive feature [4]	4, 13
APC, KRAS, TP53, SMAD4, PIK3CA	Human intestinal organoids [13]	niche signaling[13]	Grew without all niche factors, visible tumor, micrometastases [13]	13
TP53, SMAD4	adenoma organoid[13]		Chromosomal instability (CIN) phenotype, polarized morphology [13]	13

Supplementary Table 2. The mutation distribution of five genes across CRC populations

Studies	Samples	Freq.	*APC*	*TP53*	*KRAS*	*SMAD4*	*PIK3CA*
MSKCC, Genome Biol 2014	138	100%	91%	82%	55%	20%	12%
TCGA, Nature 2012	212	91.5%	76%	53%	42%	20%	14%
TCGA, Provisional	220	91.4%	73%	55%	44%	15%	15%
Genentech, Nature 2012	72	89%	40%	56%	51%	31%	24%
DFCI, Cell Reports 2016	619	77%	27%	43%	28%	21%	10%

Supplementary Table 3. The number of engineered organoids and CRC samples

Mutation type	organoids	CRC
A	5	54
AK	1	40
AS	1	3
AKST	2	1
AKSTP	1	5

Note: A-organoid with APC mutations, AK-organoid with both APC and KRAS mutations, AS-organoid with both APC and SMAD4 mutations, AKST-organoids with mutations of four genes including APC, KRAS, SMAD4 and TP53, AKSTP-organoid with mutations of five genes including APC, KRAS, SMAD4, TP53 and PIK3PA.

Supplementary Table 5. The significance of activity difference for functions targeted by five driver genes.

pathways / Mutation type	WNT	TGF-β	RAS-MAPK	TP53	PI3K
A	0.015	0.374	0.001	0.001	0.001
AS	0.001	0.012	0.001	0.022	0.001
AK	0.966	0.320	0.033	0.110	0.001
AKST	0.911	0.492	0.682	0.154	0.001
AKSTP	0.540	0.020	0.745	0.015	0.004

Note: WNT: hsa04310, RAS-MAPK: hsa04014-, TGF-β: hsa04350, TP53:hsa04115, PI3K:hsa04151
